# Evaluation of associations between condylar morphology, ramus height, and mandibular plane angle in various vertical skeletal patterns: a digital radiographic study

**DOI:** 10.1186/s12903-022-02365-1

**Published:** 2022-08-08

**Authors:** Gökhan Türker, Meriç Öztürk Yaşar

**Affiliations:** 1 Department of Orthodontics, Faculty of Dentistry, Mersin University, Mersin, Turkey; 2 Department of Orthodontics, Faculty of Dentistry, Erciyes University, Kayseri, Turkey

**Keywords:** Vertical skeletal pattern, Condylar morphology, Ramus height, Mandibular plane angle, Dental panoramic radiograph

## Abstract

**Background:**

To evaluate condylar morphology, ramus height, and asymmetry indexes in patients with different vertical skeletal patterns and to determine the association between condylar and ramal measurements with the mandibular plane angle.

**Methods:**

Dental panoramic radiographs of 60 patients with different skeletal patterns were evaluated. According to the cranial base (Sella-Nasion)-mandibular plane (SN-MP) angle, the patients were divided into three groups: normal angle (NA), low angle (LA), and high angle (HA). The condylar area, condylar perimeter, condylar heights, and ramus height were measured, and the asymmetry index value of each measurement was calculated. A one-way analysis of variance as well as a post hoc Tukey and Kruskall-Wallis tests were used to determine intergroup differences. Pearson’s correlation coefficient was used to determine the relationship between all measurements and SN-MP.

**Results:**

The intergroup comparison of condylar area, condylar perimeter, condylar heights, and ramus height measurements showed that the patients in the LA group have statistically significantly greater values compared to those in the HA group. A statistically significant difference was detected between the NA and LA groups only in the condylar area measurements. There was no statistical difference only in the ramus height measurements between the NA and HA groups. Asymmetry index values of the groups were similar. The negative correlations were found between all measurements and the SN-MP angle.

**Conclusion:**

HA individuals have lower ramus heights and smaller condylar morphologies than NA and LA individuals. In addition, as the SN-MP angle increases, the condyle dimensions and ramus height decrease, and this is a clinically important finding.

## Background

Vertical skeletal growth can be affected by various factors, such as skeletal growth of the maxilla and mandible, dentoalveolar development, and functions of the tongue and lips [1, 2]. The balance between the vertical growth of the condyles and the vertical growth of the facial sutures and alveolar processes affects the direction of the mandibular rotation and growth pattern [3, 4]. Clinicians generally consider the inclination of the mandibular plane when determining the growth pattern of individuals [4]. The normal angle (normodivergent), low angle (hypodivergent) and high angle (hyperdivergent) patterns are three basic types of vertical skeletal growth patterns that are determined using the cranial base (Sella-Nasion)-mandibular plane (SN-MP) angle [1, 5].

Distinctive facial characteristics of individuals with different vertical skeletal growth patterns include differences in mandibular ramus heights and mandibular plane angles, and mandibular condyle morphology and position can be significantly affected by the posterior rotation of the mandible [6, 7]. The idea that there may be a relationship between mandibular condylar sizes, condylar position, condylar morphology, ramus height, and vertical skeletal pattern has been discussed in the literature [1, 6, 8–11]. Individuals with a high-angle pattern may have lower mandibular ramus heights than individuals with normal angle and low-angle patterns [1, 9]. Furthermore, it is stated that in addition to the vertical skeletal pattern, gender and the sagittal relationship of the maxillae and mandible may also effect on the mandibular ramus height, condyle morphology, and mandibular asymmetries [8].

Mandibular asymmetries, which can cause functional and aesthetic problems, are characterized by dimensional and morphological differences between the left and right sides of the mandible [8, 12]. Several authors have evaluated the relationship between mandibular asymmetries and temporomandibular disorders [13, 14], mandibular first molar tooth extractions [12], cleft lips and palates [15–17], unilateral and bilateral crossbites [18–20], and different skeletal patterns [1, 8, 9].

Mandibular asymmetries can be evaluated clinically or using diagnostic materials such as posteroanterior cephalometric radiographs, dental panoramic radiographs (DPRs), and cone-beam computed tomography (CBCT) records [1, 8, 9, 14–21]. However, ramal and condylar dimensions and condyle morphology can be generally evaluated on DPRs [17–19, 21–24] and CBCT [1, 8, 9, 16]. The use of CBCT in the accurate and reliable evaluation of craniofacial structures offers important advantages, such as high resolution and three dimensional (3D) imaging [8, 25]. However, the DPRs, whose disadvantages compared to CBCT include low resolution, image distortion, superposition, and magnification, are routinely used in dentistry practice because of their economical and low radiation dose [22, 26–28]. Despite the limitations of DPRs, it has been suggested that condyle morphology can be evaluated reliably and rapidly on DPRs obtained [22]. In addition, many studies in the literature have shown that vertical condylar and ramal lengths and asymmetries can be evaluated on DPRs [12, 17–19].

It is clinically important to determine the differences in the mandible dimensions and asymmetries of individuals with different skeletal characteristics in order not to describe acceptable discrepancies as pathology and to create an accurate treatment plan [8, 9]. The aim of this study is to evaluate condyle morphology, condylar dimensions, mandibular ramus height, and asymmetry indexes in young adult patients with different vertical skeletal patterns using DPRs and to determine the association between condylar and ramal measurements with the SN-MP angle. The null hypothesis assumed that there was no significant difference in condyle morphology, condylar dimensions, mandibular ramus height of Class I individuals with different vertical growth patterns, and no correlation between condylar and ramal measurements and the SN-MP angle.

## Materials and methods

### Sample

The present retrospective study was performed in the Department of Orthodontics, Erciyes University Faculty of Dentistry after being approved by the Erciyes University Clinical Research Ethics Committee (Approval no: 2020 /435). In order to determine the sample size, the condylar area (CA) measurements of the first five patients in each group were evaluated. The power analysis (G*Power version 3.1.9.4; Franz Faul, Universität Kiel, Kiel, Germany) performed using these data showed that when 20 patients (40 condylar measurements) were included in each group, a statistical difference with 90 per cent power, at a significance level of α = 0.05 and 0.69 effect size could be obtained.

The patients included in this study had (a) no previous orthodontic treatment and/or orthognathic surgery history, (b) no craniofacial deformity, such as a cleft lip-palate, (c) no dental and/or craniofacial trauma history, (d) the absence of any systemic disease and/or long-term drug use affecting bone development, (e) no history of temporomandibular joint disorders, and (f) a skeletal Class I maxillomandibular relationship according to the ANB angle (between 0 and 4°). Furthermore, patients who were found to have anterior or posterior crossbite in clinical examination were excluded from the study, and DPRs with low diagnostic quality were not evaluated in this study.

The DPRs of 60 skeletally mature patients (30 females and 30 males; mean age, 17.94 ± 1.46 years [range, 16.00–21.75 years]) with different vertical skeletal patterns who requested for orthodontic treatments. Skeletal maturation stages were evaluated using the cervical vertebrae maturation index on the lateral cephalometric radiographs [29–31]. In addition, lateral cephalometric radiographs of all patients were used to determine the SN-MP and ANB angles. The participants were divided into three groups (Low Angle [LA] ≤ 26°; 26° < Normal Angle [NA] < 38°; High Angle [HA] ≥ 38°) according to their vertical skeletal patterns, which were established using the SN-MP angle [1, 3, 5]. All the patients had a skeletal Class I relationship. The ANB angles of the NA, LA, and HA groups were 2.18 ± 1.09°, 1.98 ± 1.32° and 2.39 ± , 1.11° respectively. The sample included 20 patients in the NA group (10 females and 10 males; mean age: 18.19 ± 2.00 years, SN-MP°: 31.01 ± 3.01°), 20 patients in the LA group (10 females and 10 males; mean age: 17.63 ± 1.12 years, SN-MPº: 24.37 ± 1.90°), and 20 patients in the HA group (10 females and 10 males; mean age: 18.01 ± 1.09 years, SN-MP°: 40.68 ± 2.45°) (Table 1).

**Table 1:** Chronological age, gender, ANB angle and SN-MP angle distribution in groups

Groups	Gender	*N*	Age (year) (mean ± SD)	ANB angle (°) (mean ± SD)	SN-MP angle (°)(mean ± SD)
Normal angle	Female	10	18.40 ± 2.11	2.12 ± 1.26	30.54 ± 2.84
	Male	10	17.98 ± 1.97	2.24 ± 0.96	31.49 ± 3.25
	Total	20	18.19 ± 2.00	2.18 ± 1.09	31.01 ± 3.01
Low angle	Female	10	17.66 ± 1.29	1.91 ± 1.29	24.70 ± 1.98
	Male	10	17.62 ± 0.98	2.04 ± 1.42	24.03 ± 1.86
	Total	20	17.63 ± 1.12	1.98 ± 1.32	24.37 ± 1.90
High angle	Female	10	18.20 ± 0.87	2.46 ± 1.10	40.50 ± 1.73
	Male	10	17.82 ± 1.29	2.33 ± 1.18	40.87 ± 3.09
	Total	20	18.01 ± 1.09	2.39 ± 1.11	40.68 ± 2.45
Total	60	17.94 ± 1.46	2.18 ± 1.17	32.02 ± 7.18	

*N* Number of subjects, SD standard deviation

### Dental panoramic radiographs and image analysis

All the DPRs were taken using the same DPR device (OP200D; Instrumentarium Dental, Tuusula, Finland; 66– 85 kVp, 10–16 mA, 14.1-s exposure time). While obtaining the DPRs, the sagittal plane was aligned with the vertical line produced by the device in accordance with the manufacturer’s recommendations, and the patients were positioned so that the Frankfurt horizontal plane was parallel to the floor.

With the measurements made on the DPRs using AutoCAD 2014 software (Autodesk Inc., San Rafael, CA, USA), data on the condylar and ramal dimensions were obtained. The landmarks and measurements based on the previous studies [21, 22] were determined as follows:

O_1_ and O_2_ points: The most lateral points of the ramus.

Ramus tangent (RT): A tangential line connecting the O_1_ and O_2_ points.

Perpendicular line 1 (PL1): The line passing through the deepest point of the sigmoid notch and perpendicular to RT.

Perpendicular line 2 (PL2): The line passing 0.25 mm above PL1 and perpendicular to RT.

Perpendicular line 3 (PL3): The line passing through the most superior point on the condyle and perpendicular to RT.

**Fig. 1:**
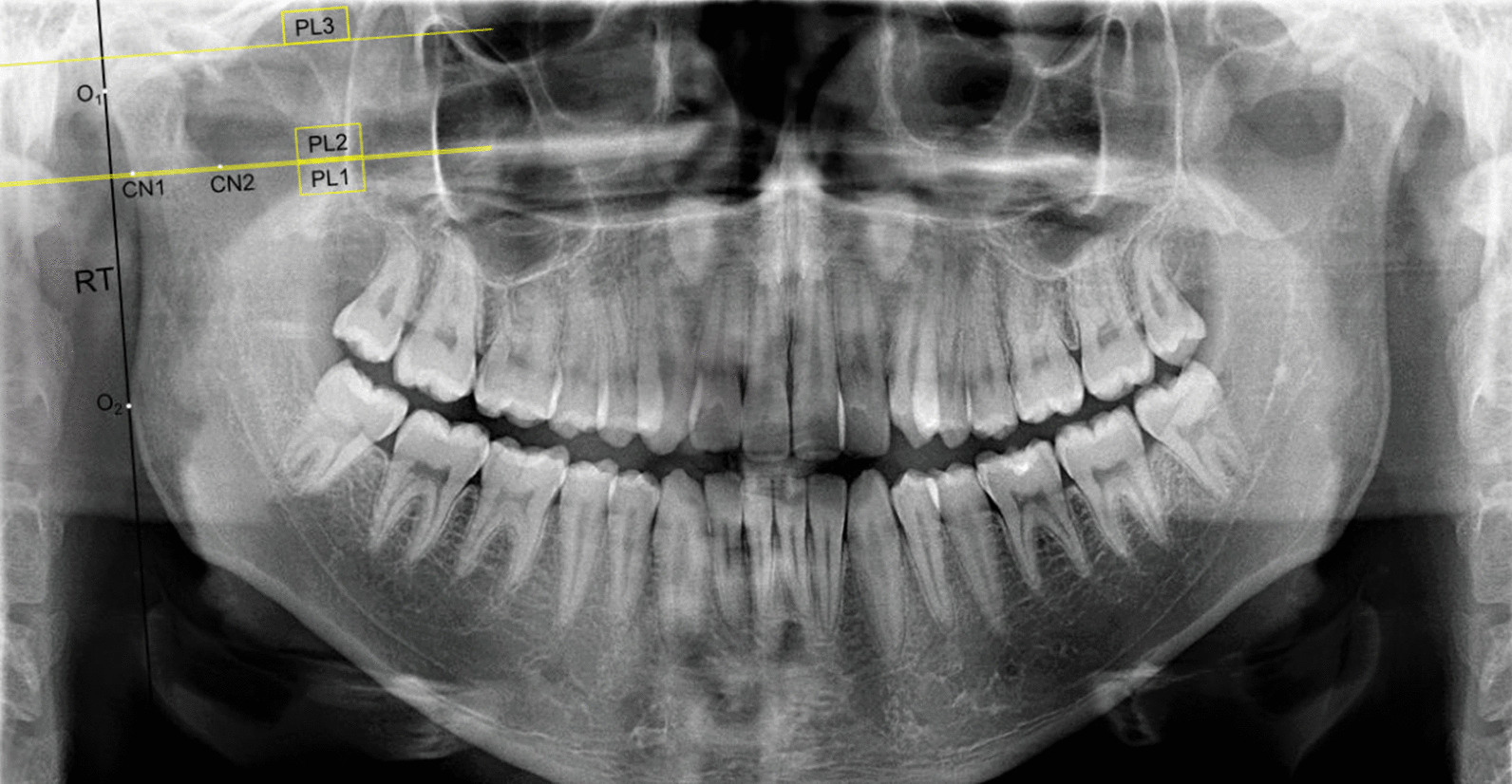
The points and lines used for measurements. O1 and O2 points: The most lateral points of the ramus, RT: Ramus tangent, PL1: Perpendicular line 1, PL2: Perpendicular line 2, PL3: Perpendicular line 3, CN1 and CN2 points: The intersections between the PL2 and the posterior (CN1) and anterior (CN2) of the condylar neck

CN1 and CN2 points: The intersections between the PL2 and the posterior (CN1) and anterior (CN2) of the condylar neck (Fig. 1).

Condylar Area (CA): The condyle area limited by the C1 and C2 line segment.

Condylar Perimeter (CP): The condyle perimeter limited by the C1 and C2 line segment.

Condylar Height 1 (CH1): The distance between the PL2 and PL3.

Condylar Height 2 (CH2): The vertical distance from the PL3 on the RT to the O_1_ point projected on the RT.

Ramal Height (RH): The distance between the O_1_ and O_2_ points on the RT.

**Fig. 2:**
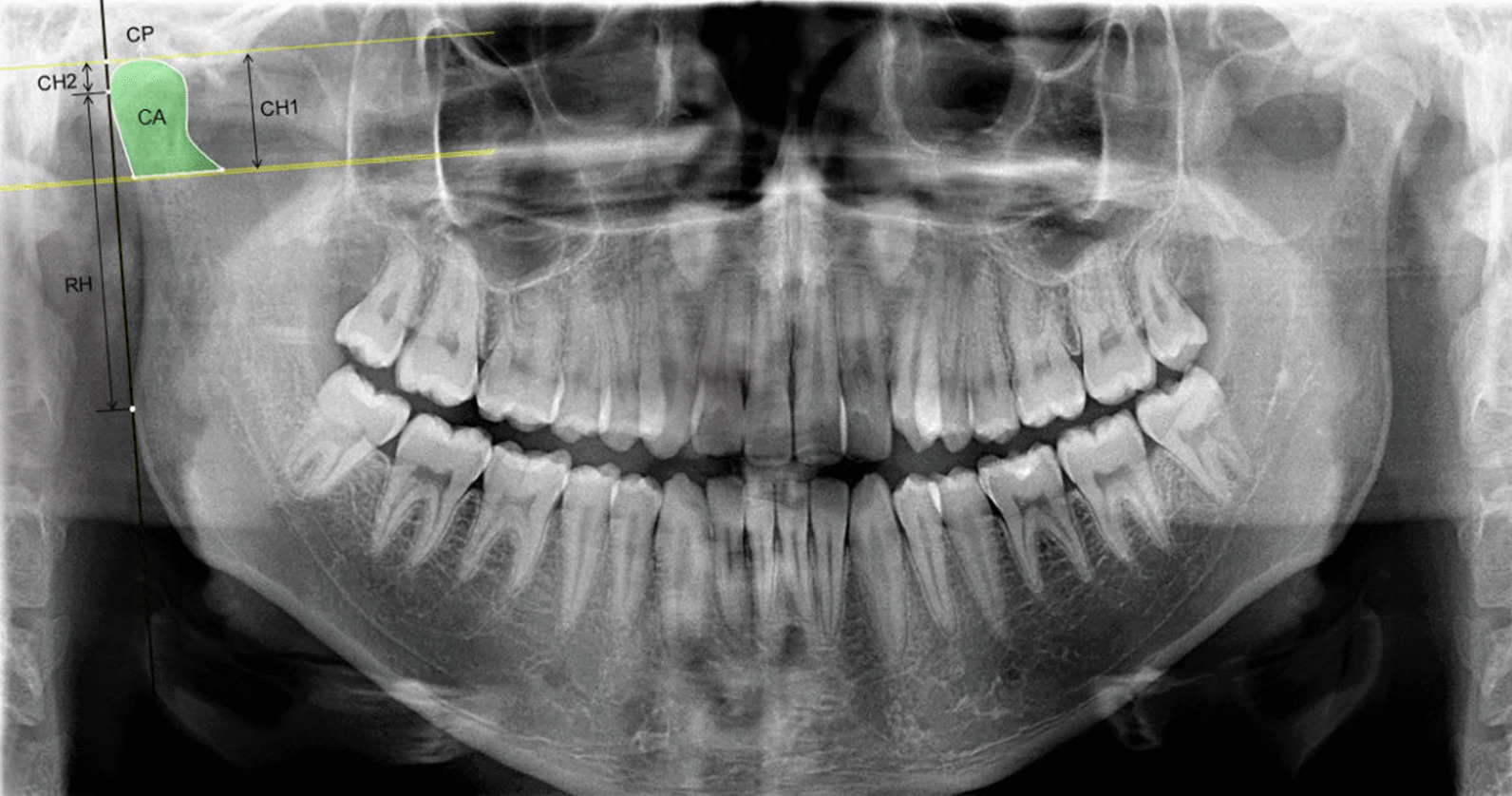
Dental panoramic radiograph showing the selection of the condylar area (CA), the condylar perimeter (CP), the condylar height 1 (CH1), the condylar height 2 (CH2), the ramal height (RH) and the total height (CRH = CH2 + RH)

Total Height (CRH): The vertical distance from the PL3 on the RT to the O_2_ point projected on the RT (Fig. 2).

The condylar area, condylar perimeter, CH1, CH2, RH, and CRH values were measured on both the right and left sides of the mandible, and the following formula was used to calculate the asymmetry indexes of all variables [21]:

$${\text{Asymmetry index }} = \, \frac{{\left( {{\text{right}} - {\text{left}}} \right)}}{{\left( {{\text{right }} + {\text{ left}}} \right)}} \, \times { 1}00$$

### Statistical analysis

To determine intraobserver reliability, the digital DPRs of the 30 patients were re-evaluated after 4 weeks, and the condylar and ramal measurements of these patients were repeated by the same investigator. The intraobserver reliability was determined by calculating the intraclass correlation coefficients (ICCs). In addition, the systematic differences between the initial and repeated measurements were evaluated using paired-samples t-test.

All data were analyzed using the Statistical Package for the Social Sciences software (SPSS version 24.0 Inc, Chicago, IL, USA). The arithmetic mean and standard deviation were calculated for each variable. The level of significance in comparisons was considered at *p* < 0.05.

The Kolmogorov–Smirnov normality test was used to evaluate the normal distribution of the data. The paired samples t-test was used to determine possible statistically significant differences between the right and left sides for the condylar and ramal measurements in each group. The independent samples t-test was carried out for comparisons between genders. A one-way analysis of variance (ANOVA) test was performed to determine differences among groups and a post hoc Tukey honestly significant difference (HSD) test was used for multiple comparisons. The Kruskall-Wallis test was performed to detect possible statistically significant differences among the group for asymmetry index measurements. Pearson’s correlation coefficient (PCC) was used to evaluate the relationship among condylar measurements, ramal measurements, and the SN-MP angle.

## Results

The ICCs were found to be within a range of 0.907–0.991 for condylar and ramal measurements and to yield high reliability for these measurements. Furthermore, the paired-samples t-test used to identify systematic differences between the initial and repeated measurements showed that the differences were insignificant.

The intragroup comparison of all measurements for the right and left sides in the NA, LA, and HA groups did not show any statistically significant difference (Table 2). According to the independent samples t-test, no statistically significant gender-related difference was found for any condylar and ramal measurements (Table 3). Therefore, the data for both sides and both genders were pooled for further statistical evaluations.

**Table 2:** Intragroup comparisons of condylar and ramal measurements for the right and left sides

Measurements	Gender	Normal angle			Low angle			High angle		
		Right side *(*mean ± SD)	Left side (mean ± SD)	*p* value	Right side *(*mean ± SD)	Left side (mean ± SD)	*p* value	Right side *(*mean ± SD)	Left side (mean ± SD)	*p* value
CA (mm^2^)	Female	202.39 ± 30.36	201.72 ± 32.46	0.944	208.57 ± 35.39	226.10 ± 43.83	0.091	171.13 ± 32.61	163.95 ± 32.48	0.355
	Male	205.85 ± 46.44	202.07 ± 46.23	0.692	241.11 ± 46.03	226.70 ± 40.85	0.170	169.62 ± 36.74	180.19 ± 52.54	0.212
	Total	204.12 ± 38.23	201.90 ± 38.88	0.732	224.84 ± 43.31	226.40 ± 41.23	0.836	170.38 ± 33.82	172.07 ± 43.32	0.766
CP (mm)	Female	62.80 ± 5.86	62.03 ± 5.01	0.662	62.54 ± 5.35	64.46 ± 7.37	0.314	57.74 ± 6.02	56.41 ± 6.04	0.354
	Male	62.68 ± 8.21	62.30 ± 7.43	0.832	67.74 ± 7.46	64.65 ± 6.11	0.085	57.69 ± 5.60	58.91 ± 8.37	0.529
	Total	62.74 ± 6.94	62.16 ± 6.17	0.633	65.14 ± 6.86	64.55 ± 6.59	0.657	57.72 ± 5.66	57.66 ± 7.22	0.961
CH1 (mm)	Female	19.26 ± 2.50	19.29 ± 2.11	0.936	19.09 ± 2.26	19.91 ± 2.91	0.122	17.53 ± 2.84	17.74 ± 2.84	0.706
	Male	19.07 ± 3.20	19.11 ± 3.23	0.953	20.48 ± 2.82	19.99 ± 2.23	0.444	17.34 ± 2.74	18.11 ± 2.63	0.096
	Total	19.17 ± 2.80	19.20 ± 2.66	0.922	19.78 ± 2.59	19.95 ± 2.52	0.684	17.44 ± 2.57	17.92 ± 2.67	0.156
CH2 (mm)	Female	7.84 ± 1.25	7.98 ± 1.24	0.779	7.74 ± 0.92	8.17 ± 1.45	0.252	6.98 ± 0.93	6.49 ± 1.27	0.076
	Male	7.62 ± 1.76	8.11 ± 1.79	0.120	7.99 ± 1.89	8.28 ± 1.91	0.694	6.16 ± 1.50	6.56 ± 1.27	0.306
	Total	7.73 ± 1.49	8.04 ± 1.50	0.260	7.86 ± 1.45	8.22 ± 1.65	0.364	6.57 ± 1.29	6.53 ± 1.24	0.871
RH (mm)	Female	46.47 ± 2.70	46.65 ± 4.43	0.898	47.78 ± 5.01	47.97 ± 4.37	0.731	46.42 ± 2.44	46.39 ± 2.99	0.975
	Male	49.03 ± 4.79	48.89 ± 4.19	0.884	50.74 ± 3.77	49.69 ± 3.76	0.268	47.25 ± 3.30	46.26 ± 4.96	0.324
	Total	47.75 ± 4.01	47.77 ± 4.35	0.978	49.26 ± 4.58	48.83 ± 4.06	0.428	46.83 ± 2.86	46.32 ± 3.98	0.412
CRH (mm)	Female	54.31 ± 3.36	54.63 ± 4.16	0.771	55.52 ± 4.90	56.14 ± 5.03	0.228	53.39 ± 3.93	52.89 ± 3.93	0.555
	Male	56.64 ± 5.04	57.00 ± 4.25	0.655	58.72 ± 2.85	57.96 ± 3.84	0.404	53.41 ± 4.13	52.82 ± 5.16	0.600
	Total	55.48 ± 4.34	55.81 ± 4.27	0.603	57.12 ± 4.23	57.05 ± 4.45	0.897	53.40 ± 3.43	52.85 ± 4.46	0.419

Paired-samples *t*-test was used

*SD* Standard deviation, *CA* Condylar area, *CP* Condylar perimeter, *CH1* Condylar height 1, *CH2* Condylar height 2, *RH* Ramal height, *CRH* Total height

**Table 3:** Comparisons of condylar and ramal measurements between genders

Measurements	Normal angle			Low angle			High angle		
	Female (mean ± SD)	Male (mean ± SD)	*p* value	Female (mean ± SD)	Male (Mean ± SD)	*p* value	Female (mean ± SD)	Male (mean ± SD)	*p* value
CA (mm^2^)	202.06 ± 30.59	203.96** ± **45.14	0.877	217.34** ± **39.80	233.90** ± **43.00	0.214	167.54** ± **31.89	174.90** ± **44.46	0.551
CP (mm)	62.41** ± **5.32	62.49** ± **7.62	0.971	63.50** ± **6.34	66.19** ± **6.82	0.204	57.08** ± **5.91	58.30** ± **6.96	0.552
CH1 (mm)	19.28** ± **2.25	19.09** ± **3.13	0.833	19.50** ± **2.57	20.23** ± **2.49	0.363	17.63** ± **2.63	17.73** ± **2.64	0.913
CH2 (mm)	7.91** ± **1.21	7.86** ± **1.74	0.922	7.95** ± **1.21	8.13** ± **1.86	0.716	6.73** ± **1.11	6.36** ± **1.37	0.349
RH (mm)	46.56** ± **3.57	48.96** ± **4.38	0.066	47.88** ± **4.58	50.21** ± **3.70	0.084	46.40** ± **2.66	46.75** ± **4.13	0.751
CRH (mm)	54.47** ± **3.68	56.82** ± **4.54	0.080	55.83** ± **4.84	58.34** ± **3.31	0.064	53.14** ± **3.33	53.11** ± **4.56	0.984

Independent samples *t*-test was used

*SD* Standard deviation, *CA* Condylar area, *CP* Condylar perimeter, *CH1* Condylar height 1, *CH2* Condylar height 2, *RH* Ramal height, *CRH* Total height

The comparison of CA, CP, CH1, CH2, RH, and CRH measurements among the participants in the NA, LA, and HA groups using one-way ANOVA and post hoc Tukey HSD tests are shown in Table 4. While a statistically significant difference was detected only in the CA measurements between the NA and LA groups (*p* < 0.05), no significant difference was observed in the other measurements. The condylar area, condylar perimeter, CH1, CH2, and CRH measurements were significantly greater in the NA group compared to the HA group (*p* < 0.05), while there was no significant difference in the RH measurements between these groups. All measurements in the LA group were statistically significantly greater compared to the HA group (*p* < 0.05).

**Table 4:** Comparisons of condylar and ramal measurements among study groups

Measurements	Normal angle (NA) (mean ± SD)	Low angle (LA) (mean ± SD)	High angle (HA) (mean ± SD)	*p* value (Tukey HSD test)		
				NA-LA	NA-HA	LA-HA
CA (mm^2^)	203.01** ± **38.07	225.62** ± **41.75	171.22** ± **38.37	0.031*	0.001**	< 0.001***
CP (mm)	62.45** ± **6.49	64.85** ± **6.64	57.69** ± **6.40	0.231	0.004**	< 0.001***
CH1 (mm)	19.18** ± **2.69	19.87** ± **2.52	17.68** ± **2.60	0.474	0.030*	0.001**
CH2 (mm)	7.89** ± **1.48	8.04** ± **1.55	6.55** ± **1.25	0.880	< 0.001***	< 0.001***
RH (mm)	47.76** ± **4.13	49.04** ± **4.28	46.58** ± **3.43	0.318	0.380	0.017*
CRH (mm)	55.65** ± **4.25	57.09** ± **4.29	53.13** ± **3.94	0.273	0.021*	< 0.001***

One-way ANOVA and post hoc Tukey HSD tests were used. Statistical significance degree: * *p* < 0.05, ** *p* < 0.01, *** *p* < 0.001

The results of the statistical analysis showed that the asymmetry index measurements of the CA, CP, CH1, CH2, RH, and CHR were not statistically different among the NA, LA, and HA groups (Table 5).

**Table 5:** Comparisons of asymmetry indexes among the study groups

Asymmetry Index	Normal angle (*N*:20)		Low angle (*N*:20)		High angle (*N*:20)		*p* value
	mean ± SD	median (min–max)	mean ± SD	median (min–max)	mean ± SD	median (min–max)	
CAI	5.87** ± **4.08	6.14 (0.01–13.26)	5.70** ± **4.13	4.89 (0.05–15.08)	5.98** ± **3.91	5.32 (0.31–16.75)	0.957
CPI	3.55** ± **2.23	3.18 (0.32–7.87)	3.14** ± **2.97	1.96 (0.31–11.61)	3.04** ± **2.99	1.87 (0.11–10.06)	0.382
CH1I	3.43** ± **2.34	3.28 (0.00–9.29)	3.45** ± **2.73	2.40 (0.02–10.02)	3.59** ± **2.47	3.08 (0.08–8.60)	0.924
CH2I	6.72** ± **4.41	6.67 (0.26–16.38)	7.13** ± **6.73	4.73 (0.20–27.45)	6.43** ± **4.70	5.94 (0.04–16.63)	0.937
RHI	2.85** ± **2.09	2.95 (0.06–9.57)	1.92** ± **1.39	1.48 (0.18–4.77)	2.43** ± **1.67	2.28 (0.28–7.15)	0.329
CRHI	1.87** ± **1.61	1.73 (0.00–6.93)	1.60** ± **1.07	1.59 (0.05–3.61)	2.34** ± **1.57	2.55 (0.44–5.98)	0.314

Kruskall-Wallis test was used

*N* number of subjects, *SD* Standard deviation *CAI* Condylar area asymmetry index, *CPI* Condylar perimeter asymmetry index, *CH1I* Condylar height 1 asymmetry index, *CH2I* Condylar height 2 asymmetry index, *RHI* Ramal height asymmetry index, *CRHI* Total height asymmetry index

The results of the correlation analysis showing the relationship between condylar and ramal measurements as well as the relationship between these measurements and the SN-MP angle are provided in Table 6. There was a negative correlation between the SN-MP angle and the CA, CP, CH1, CH2, and CRH measurements at the *p* < 0.01 significance level, while the negative correlation between the SN-MP and RH dimension was at the *p* < 0.05 significance level. There was no significant correlation between CH2 and RH dimensions, and positive statistically significant correlations were found between all other condylar and ramal measurements (*p* < 0.01).

**Table 6:** Relationships among all condylar and ramal parameters and the SN-MP angle

Measurements	SN− MP (°)	CA (mm^2^)	CP (mm)	CH1 (mm)	CH2 (mm)	RH (mm)	
CA (mm^2^)	*r* value	− 0.501**					
	*p* value	< 0.001					
CP (mm)	*r* value	− 0.425**	0.948*				
	*p* value	< 0.001	< 0.001				
CH1 (mm)	*r* value	− 0.346**	0.886**	0.912**			
	*p* value	< 0.001	< 0.001	< 0.001			
CH2 (mm)	*r* value	− 0.399**	0.504**	0.475**	0.483**		
	*p* value	< 0.001	< 0.001	< 0.001	< 0.001		
RH (mm)	*r* value	− 0.196*	0.356**	0.341**	0.328**	0.064	
	*p* value	0,032	< 0.001	< 0.001	< 0.001	0.489	
CRH (mm)	*r* value	− 0.320**	0.503**	0.480**	0.470**	0.412**	0.936**
	*p* value	< 0.001	< 0.001	< 0.001	< 0.001	< 0.001	< 0.001

r value: Pearson’s correlation coefficient (PCC), Correlation significance level: * *p* < 0.05, ** *p* < 0.01

*CA* Condylar area, *CP* Condylar perimeter, *CH1* Condylar height 1, *CH2* Condylar height 2, *RH* Ramal height, *CRH* Total height

## Discussion

The morphology, dimensions, and symmetry of craniofacial structures such as the mandibular condyle and ramus can be affected by certain systemic diseases, craniofacial anomalies, the sagittal and transversal relationship of the maxillae and mandible, and vertical skeletal patterns [1, 8, 9, 16–19, 23]. Although controversial, the results of studies evaluating ramus length, condyle dimensions, condylar distances, and asymmetries in individuals with a different vertical skeletal pattern have generally asserted that hyperdivergent individuals have lower ramus length values [1, 8, 9]. Determining whether there is a correlation between vertical skeletal parameters such as SN-MP and condyle and ramus dimensions may contribute to this research. In this study, the condylar morphology, ramus lengths, and asymmetries were evaluated in individuals with different vertical patterns using CA, CP, CH1, CH2, RH, and CRH measurements on DPRs. In addition, it aimed to provide information about the relationship of these measurements with the SN-MP angle.

A two-dimensional (2D) or 3D radiographic examination can be used to evaluate the dimensions and morphology of the mandibular condyle and ramus [1, 8, 9, 17–19, 22–24]. Although CBCT is considered the gold standard for this type of craniofacial examination and provides more diagnostic information [1, 9], its radiation dose is higher than DPRs, and thus it is costly [24]. DPRs offer several advantages: they can be used routinely in dental examinations, are low-cost, and expose patients to relatively low doses of radiation [12]. It has been stated that vertical measurements on DPRs are acceptably repeatable provided that the patient’s head is properly positioned [32]. Momjian et al. reported that the condylar area, condylar perimeter, and condylar height could be reliably calculated on DPRs [22]. In addition, condylar and ramal heights were used in 2D evaluations to determine asymmetries [12, 17–19, 21]. Low-cost and easily accessible diagnostic materials such as DPRs can be used for simple evaluation of morphological differences in different skeletal patterns in radiological diagnoses, orthodontic treatments and orthognathic surgery planning. In the present study, DPRs with adequate diagnostic quality were used; these were taken when the head of each patient was in the ideal position, especially considering the radiation dose.

In our study, the comparison of right and left sides for the CA, CP, CH1, CH2, RH, and CRH values in the NA, LA, HA groups did not show a significant difference, indicating that condylar morphology, condylar heights, and mandibular ramus height were symmetrical in all groups. Furthermore, there was no statistical difference between the genders in the intragroup comparisons. Similarly, some studies [12, 17–19] reported that there was no statistically significant difference in condyle (CH2), ramus (RH), and total (CHR) heights for the right and left sides in patients without crossbites, tooth extractions, and cleft lips and palates. In addition, the posterior mandibular vertical measurements [17, 18] and asymmetries [12, 19] on DPRs in individuals with normal occlusion were generally similar between genders. In a study that compared the CH2, RH, and CRH measurements in patients with different vertical patterns, it was reported that there was a slight difference in CH2 values between the right and left sides in only low angle individuals [1]. The 3D evaluations performed in adult patients with different skeletal classes and vertical patterns showed that there was no difference in CH2, RH, CRH, and condyle volume between the right and left sides [8].

The vertical skeletal pattern can affect condylar dimensions, condylar morphology, condylar position, and ramus length [8–11]. The present study evaluated all condylar and ramal parameters in the NA, LA, HA groups, which were determined according to the SN-MP angle, and showed that these parameters had lower values in individuals with HA vertical skeletal patterns. In addition, although all parameters were higher in individuals with LA vertical skeletal patterns compared to the other groups, only the condylar area was statistically significantly higher than the NA group. These findings are consistent with the claim that a lower ramus height can be seen especially in individuals with HA vertical skeletal pattern [1]. Similarly, in studies showing that individuals with HA skeletal patterns tend to have smaller condylar sizes and more superiorly positioned condyles than those with LA skeletal patterns, it was stated that condylar morphology and position may vary according to the vertical facial morphology [10, 11]. In a recent study, Lemes et al. [9] reported that individuals with a hyperdivergent skeletal pattern had significantly shorter mandibular ramus heights, in comparison to those with normodivergent and hypodivergent skeletal patterns. They also suggested that changes in the mandibular plane angle, as well as in the other horizontal planes, rather than the mandibular ramus length, would have a greater contribution to vertical facial discrepancies [9]. Our study determined that all parameters involved in ramus height have a negative correlation with the SN-MP angle. In this study, it was determined that all parameters related to ramus height and condylar morphology had a negative correlation with the SN-MP angle. In addition, it was determined that there was no correlation only between CH2 and RH but positive correlations between other condylar and ramal measurements. Therefore, correlation findings showing relationships between SN-MP angle and condylar morphology and ramus height suggested that vertical skeletal discrepancies might be affected by condylar dimensions and ramus heights. The main clinical implication suggested by the present results is that variations in condylar morphology and ramus height may be the result of changes in SN-MP angle in individuals without temporomandibular joint disorders and with normal sagittal and transversal maxillomandibular relationships.

Regarding the asymmetry index values of CA, CP, CH1, CH2, RH, and CRH, no statistically significant differences were present among the NA, LA, and HA groups. Also, differences between right and left sides were not statistically significant for each group. When the effects of the vertical pattern on condylar and ramal asymmetries were examined, it was found that more asymmetries could be seen in hyperdivergent individuals [8], and it was also suggested that ramus length asymmetry indices were similar in individuals with different vertical patterns [1, 9]. Our findings support the view that the vertical skeletal pattern has no effect on condylar and ramal asymmetries. In addition, considering the asymmetry index values of CA and CP, it was observed that the vertical skeletal pattern did not affect condylar morphological asymmetries.

In our study, unlike other studies [1, 8–11], the preference for the DPR evaluation over the CBCT evaluation is a limitation that must be acknowledged. It is stated that CBCT has advantages such as higher accuracy and reliability over DPRs in determining mandibular posterior vertical asymmetry [33]. Although CBCT is a more advanced imaging method, ethical limitations made it preferable to evaluate DPRs, as they deliver a less harmful dose of ionized radiation. Furthermore, in recent studies [34, 35], it is seen that DPRs are also used in radiological evaluations such as fractal analysis and measurement of ramus dimensions. Based on the ALARA (As Low As Reasonably Achievable) principle, a dose of ionized radiation that is unlikely to improve treatment outcomes should be considered excessive, regardless of how low it is [36]. The individuals included in our study did not have any craniofacial anomalies or malocclusions that could contribute to the diagnosis by 3D imaging evaluations were made on young adult individuals. It has been shown that the use of CBCT in orthodontic treatments significantly increases the radiation dose compared to conventional methods such as DPRs and causes a higher radiation risk especially in children and adolescents compared to adults [37]. Therefore, evaluations have been made on DPRs that provide less ionized radiation than CBCT, are routinely used in dentistry, and can allow rapid and reliable assessments of posterior mandibular morphology [22]. SN-MP angle, SN-Gonion-Gnathion plane (SN-GoGn) angle, Frankfort mandibular plane angle (FMA), Y axis can be used to evaluate vertical skeletal growth of individuals [3, 38, 39]. The determination of different vertical skeletal patterns using only the SN-MP angle can be considered as another limitation in our study. Ahmed et al. [39] reported that there was a strong positive correlation between the SN-MP and SN-GoGn angles, and that SN-GoGn and FMA were the most reliable indicators for evaluating the vertical growth pattern. However, another limitation of the present study is the evaluation of vertical skeletal pattern differences in patients with only Class I sagittal relationships between the maxilla and mandible. Further studies can be conducted with participants who have skeletal Class II and Class III sagittal relationships.

## Conclusion

The null hypothesis was rejected. In each group consisting of individuals with different vertical skeletal patterns, condyle morphology and ramus height did not differ between genders. In addition, clinically important results were obtained showing that individuals with HA vertical skeletal pattern had lower ramus height and smaller condylar morphology, and condylar dimensions and ramus height decreased as the SN-MP angle increased. The asymmetry index values of condylar dimensions and ramus height do not vary according to the vertical skeletal pattern. Dentists and surgeons should be aware of the condylar and ramal morphological differences that may occur due to the SN-MP angle when evaluating temporomandibular joint disorders and planning surgical correction of malocclusions.

## Data Availability

The data that support the findings of this study are available from the corresponding author upon reasonable request.

## Abbreviations

DPRs: Dental panoramic radiographs
NA: Normal angle
LA: Low angle
HA: High angle
CBCT: Cone beam computed tomography
RT: Ramus tangent (RT)
PL1: Perpendicular line 1 (PL1)
PL2: Perpendicular line 2
PL3: Perpendicular line 3
CN1: Posterior condylar neck point
CN2: Anterior condylar neck point
CA: Condylar area
CP: Condylar perimeter
CH1: Condylar height 1
CH2: Condylar height 2
RH: Ramal height
CRH: Total height
ICCs: Intraclass correlation coefficients
ANOVA: A one-way analysis of variance
HSD: Honestly significant difference
PCC: Pearson’s correlation coefficient
