# Correction: Evaluation of associations between condylar morphology, ramus height, and mandibular plane angle in various vertical skeletal patterns: a digital radiographic study

**DOI:** 10.1186/s12903-022-02396-8

**Published:** 2022-08-30

**Authors:** Gökhan Türker, Meriç Öztürk Yaşar

**Affiliations:** 1 Department of Orthodontics, Faculty of Dentistry, Mersin University, Mersin, Turkey; 2 Department of Orthodontics, Faculty of Dentistry, Erciyes University, Kayseri, Turkey

## Correction to: Türker and Öztürk Yaşar BMC Oral Health (2022) 22:330 10.1186/s12903-022-02365-1

In the original version of this article, in the bottom row of Table 1, the columns are shifted to the left. Also, in the top row of Table 6, the columns are shifted to the left. The corrected Tables 1 and 6 are given below.

**Table 1:** Chronological age, gender, ANB angle and SN-MP angle distribution in groups

Groups	Gender	*N*	Age (Year) (Mean ± SD)	ANB Angle (°) (Mean ± SD)	SN-MP Angle (°) (Mean ± SD)
Normal angle	Female	10	18.40 ± 2.11	2.12 ± 1.26	30.54 ± 2.84
	Male	10	17.98 ± 1.97	2.24 ± 0.96	31.49 ± 3.25
	Total	20	18.19 ± 2.00	2.18 ± 1.09	31.01 ± 3.01
Low angle	Female	10	17.66 ± 1.29	1.91 ± 1.29	24.70 ± 1.98
	Male	10	17.62 ± 0.98	2.04 ± 1.42	24.03 ± 1.86
	Total	20	17.63 ± 1.12	1.98 ± 1.32	24.37 ± 1.90
High angle	Female	10	18.20 ± 0.87	2.46 ± 1.10	40.50 ± 1.73
	Male	10	17.82 ± 1.29	2.33 ± 1.18	40.87 ± 3.09
	Total	20	18.01 ± 1.09	2.39 ± 1.11	40.68 ± 2.45
Total		60	17.94 ± 1.46	2.18 ± 1.17	32.02 ± 7.18

*N* number of subjects, *SD* standard deviation

**Table 6:** Relationships among all condylar and ramal parameters and the SN-MP angle

Measurements		SN-MP (°)	CA (mm^2^)	CP (mm)	CH1 (mm)	CH2 (mm)	RH (mm)
CA (mm^2^)	*r* value	− 0.501**					
	*p* value	< 0.001					
CP (mm)	*r* value	− 0.425**	0.948**				
	*p* value	< 0.001	< 0.001				
CH1 (mm)	*r* value	− 0.346**	0.886**	0.912**			
	*p* value	< 0.001	< 0.001	< 0.001			
CH2 (mm)	*r* value	− 0.399**	0.504**	0.475**	0.483**		
	*p* value	< 0.001	< 0.001	< 0.001	< 0.001		
RH (mm)	*r* value	− 0.196*	0.356**	0.341**	0.328**	0.064	
	*p* value	0.032	< 0.001	< 0.001	< 0.001	0.489	
CRH (mm)	*r* value	− 0.320**	0.503**	0.480**	0.470**	0.412**	0.936**
	*p* value	< 0.001	< 0.001	< 0.001	< 0.001	< 0.001	< 0.001

r value: Pearson’s correlation coefficient (PCC), Correlation significance level: *p < 0.05, **p < 0.01

*CA* Condylar area, *CP* Condylar perimeter, *CH1* Condylar height 1, *CH2* Condylar height 2, *RH* Ramal height, *CRH* Total height

The original article has been corrected.

